# Understanding Implementation and Improving Nutrition Interventions: Barriers and Facilitators of Using Data Strategically to Inform the Implementation of Maternal Nutrition in Uttar Pradesh, India

**DOI:** 10.1093/cdn/nzab081

**Published:** 2021-06-02

**Authors:** Melissa F Young, Ahad Bootwala, Shivani Kachwaha, Rasmi Avula, Sebanti Ghosh, Praveen Kumar Sharma, Vishal Dev Shastri, Thomas Forissier, Purnima Menon, Phuong H Nguyen

**Affiliations:** Hubert Department of Global Health, Emory University, Atlanta, GA, USA; Hubert Department of Global Health, Emory University, Atlanta, GA, USA; International Food Policy Research Institute, Washington, DC, USA; International Food Policy Research Institute, Washington, DC, USA; FHI360, Washington, DC, USA; FHI360, Washington, DC, USA; FHI360, Washington, DC, USA; FHI360, Washington, DC, USA; International Food Policy Research Institute, Washington, DC, USA; International Food Policy Research Institute, Washington, DC, USA

**Keywords:** maternal nutrition, implementation science, qualitative research, data use, India

## Abstract

**Background:**

In response to the high levels of maternal nutrition in Uttar Pradesh, Alive & Thrive (A&T) aimed to strengthen the delivery of nutrition interventions through the government antenatal care platform, including leveraging ongoing data collection to improve program delivery and reach (registered at clinicaltrials.gov as NCT03378141). However, we have a limited understanding of providers’ experiences and challenges in collecting and using data for decision making.

**Objective:**

The aim was to identify barriers and facilitators to the *1*) collection of data and *2*) use of data for decision making.

**Methods:**

In-depth interviews (*n* = 35) were conducted among block-level government staff, frontline worker (FLW) supervisors, and A&T staff in 2 districts in Uttar Pradesh. Systematic coding of verbatim transcripts and detailed summaries were undertaken to elucidate themes related to data collection and use. FLW supervisors (*n* = 103) were surveyed to assess data use experiences.

**Results:**

Data were used to understand the reach of maternal nutrition services, estimate the demand for supplements, and guide identification of areas of low FLW performance. About half of supervisors reported using data to identify areas of improvement; however, only 23% reported using data to inform decision making. Facilitators of data collection and use included collaboration between health department officials, perceived importance of block ranking, and monthly review meetings with staff and supervisors to review and discuss data. Barriers to data collection and use included human resource gaps, inadequate technology infrastructure, FLW educational level, political structure, and lack of cooperation between FLWs and supervisors.

**Conclusions:**

The use of data for decision making is critical for supporting intervention planning and providing targeted supervision and support for FLWs. Despite intensive data-collection efforts, the use of data to inform decision making remains limited. Collaboration facilitated data collection and use, but structural barriers such as staff vacancies need to be addressed to improve the implementation of maternal nutrition interventions.

## Teaser

Despite intensive data-collection efforts, the use of data to inform decision making remains limited. Structural barriers such as staff vacancies need to be addressed to improve data utilization.

## Introduction

High rates of maternal mortality and malnutrition remain a critical global health problem and key priority area for meeting Sustainable Development Goals (1, 2). While there are clear evidence-based interventions to improve maternal nutrition (3–5), global progress has been inadequate. There remain critical evidence gaps on how to effectively improve the implementation of maternal nutrition interventions at scale (6, 7). Furthermore, there are large disparities in the delivery of essential nutrition and health services, often not reaching those with the most need (8–10).

Globally, there have been calls for data-driven accountability to identify problems and vulnerable populations, prioritize actions, monitor progress, and improve program implementation (6, 11–13). Effective use of data may be used to identify critical bottlenecks and facilitators for the implementation of nutrition interventions, and improve program delivery and impact (14). For example, India has demonstrated a successful use of routine program data by service providers for Polio and HIV/AIDS programs, which led to program improvements that effectively translated into services reaching targeted groups (15). While there has been growing recognition and utilization of a data-driven approach to inform the implementation of nutrition interventions and programs (16–19), this has not been universal and further guidance is needed on best practices for embracing a data-driven approach. There remain critical questions on key factors that could inhibit or facilitate data-collection and utilization efforts within the context of ongoing nutrition interventions.

In order to address this gap, we conducted an in-depth case study to examine the key factors that influence data collection and use in an ongoing maternal nutrition intervention reaching ∼16,000 women over 12 mo in Uttar Pradesh, India (20). Despite political support and the presence of progressive policies prioritizing maternal nutrition in India, a streamlined package of nutritional services is not reaching the majority of women during pregnancy (10, 21). In Uttar Pradesh, only 26% of women attend 4 or more antenatal care visits and 13% of women receive the recommended number of iron and folic acid (IFA) supplements during their pregnancy (22). In this setting, 1 in 4 women are underweight and over half of pregnant women are anemic, placing women and their children at risk for poor birth outcomes (2, 22–24). To address the challenges of high levels of maternal malnutrition and low coverage of health and nutrition interventions, Alive & Thrive (A&T), in collaboration with the Government of Uttar Pradesh, implemented a project to strengthen integration of Maternal Nutrition Interventions in Existing Reproductive, Maternal, Neonatal, Child, and Adolescent Health Services. The package of interventions included social mobilization, training of frontline workers (FLWs), strengthening supportive supervision mechanisms, strengthening maternal nutrition services, supply chain management, and strategic use of data. The strengthening of data-driven decision making to improve services at cluster subcenter and block levels comprised the following: *1*) capacity building of government Health and Integrated Child Development Services (ICDS) supervisors on strategic use of data; *2*) support to conduct data-driven meetings; *3*) adoption of a monthly report card based on routine government monitoring data on key maternal nutrition indicators to facilitate the data-driven review process; and *4*) adoption during health review meetings of the use of a dashboard visualizing data on routine government monitoring, stock availability, and supportive supervision. While an impact evaluation has assessed the overall impact of the intervention package on maternal nutrition practices (25), there is a critical gap in our understanding of key factors that influenced the strategic use of data. This study aims to identify barriers and facilitators to the *1*) collection of data and *2*) use of data for decision making.

## Methods

### Setting

The larger study takes place in 26 blocks from the 2 study districts in Uttar Pradesh, India. These blocks are mainly representative of rural areas, with the majority of pregnant women being housewives (90%), with a low education (only a third of women had completed high school or above), belonging to disadvantaged classes (>80% were scheduled castes/other backward classes), and 17% being food insecure (25). Additional details of the larger study context and primary intervention have been described in detail elsewhere (25).

### Study design

A mixed-methods study was conducted in collaboration among Emory University, International Food Policy Research Institute (IFPRI), and A&T. We conducted in-depth interviews (*n* = 35) between July and August 2019 and a survey (*n* = 103) in December 2019. This project was approved by the institutional review boards of Emory University (qualitative study, IRB00111064) and IFPRI (quantitative study, registered as NCT03378141) in the United States and the Committee for Scientific Review and Evaluation of Biomedical Research in India.

### Qualitative data

We conducted in-depth interviews with block-level government staff, FLW supervisors, and A&T staff in 2 districts of Uttar Pradesh: Unnao and Kanpur-Dehat (**Supplemental Figure 1**). From each district, 1 high-performing and low-performing block was purposively sampled. The categorization of high- and low-performing blocks was based on 12 key indicators (such as attendance of pregnant women at events, number of community events, supportive supervision, distribution of supplements, etc.). The rationale for purposive sampling of blocks by performance was to help ensure a diversity of experiences and levels of program implementation in order to identify barriers to and facilitators of data collection and use. In each block, 5 block-level staff [including 1 block coordinator, 1 block community process manager, 1 block medical officer, 1 block medical officer in-charge, and 1 child development program officer (CDPO)] and 3 FLW supervisors were conveniently sampled from existing staff lists, yielding a total sample size of 32 (20 government block staff and 12 FLW supervisors). Additionally, 3 program staff were interviewed. All staff were purposefully selected to represent a diversity of views across the program spectrum.

Semi-structured interview guides unique to each stakeholder group were designed and included questions about 3 overarching data sources used to monitor the progress of the maternal nutrition program in Uttar Pradesh: government monitoring data, intervention monitoring data, and Maternal Nutrition (MN) block cards (**Supplemental Material**). Government monitoring data consisted of monthly progress reports, Uttar Pradesh Health Management Information System and the national Health Management Information System (HMIS). Intervention monitoring data consisted of program activity sheets, quarterly household surveys, and supportive supervision checklists. MN block cards combined data from both government and intervention monitoring data to provide a quick summary on key maternal nutrition indicators.

Prior to data collection, the interview guides were pilot tested and revisions to the guides were made accordingly. After verbal consent was obtained, interviews were audio-recorded on a password-protected mobile device. All interviews were voluntary and confidential. Participants were informed that there were no consequences associated with parti-cipation and their individual responses would not be shared with their employer. Participants were free to refuse to answer any questions or decide to end the interview at any point. No compensation was provided. All interviews were conducted at participants’ respective office spaces or community/primary health centers; each lasted between 30 and 90 min. Interviews were completed in the local language or English. All interviews were transcribed with detailed field notes and interviews in the local language were translated to English. Transcripts were stored on a password-protected computer to ensure privacy.

### Quantitative data

FLW supervisors (*n* = 103) were surveyed as part of the main impact evaluation of the A&T maternal nutrition interventions (26). Surveys were conducted in all 26 intervention and control blocks across the 2 study districts (Unnao and Kanpur-Dehat). Data were collected via face-to-face interviews by local trained enumerators using structured questionnaires, which were prepared in English and translated and conducted in Hindi. Key topics of questionnaires included use of data from different platforms, challenges in using data, and exposure to and use of MN block cards and supportive supervision checklist data (Supplemental Material). Verbal informed consent was obtained from all participants before conducting the questionnaire. Participants were assured that participation was voluntary and that their identity would be kept confidential.

Enumerators were recruited locally by an experienced and well-qualified survey firm, Network for Engineering and Economics Research and Management (NEERMAN). Enumerator training focused on technical content as well as security and confidentiality issues by mixed methods (lecture, role play, mock interview, and practice) in a classroom and field settings. Field supervisors received additional training related to quality-control processes; cross-checking, editing, and coding of the questions; and security and confidentiality issues. Each interview took ∼1 h.

### Data analysis

Analysis of qualitative data was completed using the principles of thematic analysis (27). Memos were created to keep track of thought processes, link categories and themes, and brainstorm potential codes. Interview transcripts, field notes, and memos were reviewed, and inductive codes were created and defined accordingly. Deductive codes were created based on themes addressed by questions in each interview guide. Inductive and deductive codes were consolidated into 3 separate codebooks based on the target group being interviewed (1 each for program staff, block-level government staff, and FLW supervisors). Codes were categorized into 2 broad topics: data collection and data use. Microsoft Excel (Microsoft Corporation) was used to create and organize codebooks. Data coding was completed after initial review of data by using the a priori codebook on themes about the barriers and facilitators for the collection and use of data. The most telling quotes were selected, and key quotes were transcribed from Hindi to English to correspond to code definitions. Data from high- and low-performing blocks were analyzed and reported together.

Quantitative data were analyzed with SAS 9.4 (SAS Institute) to provide basic descriptive statistics (means, percentage) on the challenges and use of data in intervention communities.

## Results

### Qualitative data

**FIGURE 1:**
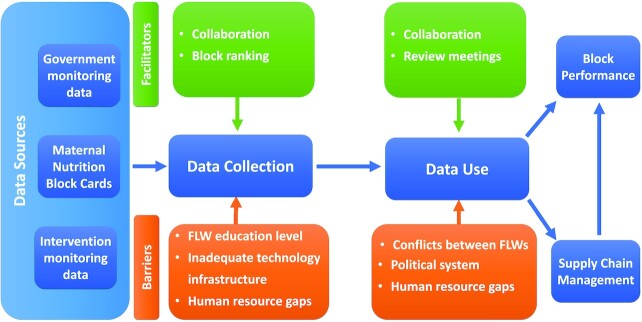
Barriers and facilitators of data use to inform the implementation of maternal nutrition interventions in Uttar Pradesh, India. Blue: Process of data flow; Green: Facilitators of data collection and use; Orange: Barriers of data collection and use. FLW, frontline worker.

The process by which data were collected and used for decision making on implementing maternal nutrition interventions is outlined in **Figure 1**. Insights from the in-depth interviews on key facilitators and barriers for data collection and data use are described below and summarized in **Table 1**.

**TABLE 1:** Summary of key themes and quotes for the barriers and facilitators of data collection and use of data in a maternal nutrition intervention in Uttar Pradesh^1^

Topic and theme	Definition	Example quotes
Collection of data		
+ Collaboration	Facilitator: Collaboration between different agencies to overcome logistical issues and support each other in timely data collection	“In meetings [with staff in other agency] sometimes my team or maybe if there is a routine immunization session and if AWC is closed and ANM has to be outside. Sometimes if AWW doesn't call children and say they won't support, then we will find solutions for this through meetings together.” - Block staff, high-performing block“In [monthly review] meeting I will tell that in 1st August you have VHND and so on and each of them note only their date of VHND." - Block staff, high-performing block
+ Block ranking	Facilitator: Motivation to achieve a high block ranking facilitates timely data collection	“If we don't fill the data on time, then our ranking will go down. We will not be at a 100%…the rank for the CHC will be low, which will affect the block and eventually the district." - Block staff, high-performing block“HMIS data is very important for us and the district. That is because the overall ranking of the district and block depends on HMIS…for example, is overall ANC good or not. Based on that, all the districts are ranked. Then the blocks are ranked.” - Block staff, low-performing block
− Suboptimal FLWeducation	Barrier: Educational level and lack of expertise in working with technical data	“The thing is that ANMs are older and have passed just 12th grade. And these [maternal nutrition indicators] are technical data on what should be done and to what extent. So now we have 50–60% of ANMs who give wrong data.” - Block staff, high-performing block“In the last fiscal year, we have done about 20 trainings for HMIS at the block level. We specifically focused on pregnant women data, like 1st trimester ANC registration…and then when we call the ANM to give the report for UP HMIS, she will ask ‘what's UP HMIS?’” - Block staff, low-performing block
− Human resourcegaps	Barrier: Staff vacancies and disruptions in the data collection workflow	"We currently have 9 vacant subcenters and 2 more will happen by July 31st…wherever there are vacancies, there will be problems with all the indicators [in the data]. The flow will not be the same as a subcenter with an ANM present.” - Block staff, low-performing block
		“We don't have other staff…no operator to compile [data]. There should be 6 supervisors and only 2 are working…supervisors have to do office work, [field] visits, operate [Anganwadi] centers, and build skills of FLWs.” - Block staff, high-performing block “We face problems because reporting is too much in our department…These days we do not have a clerk so we have to see the treasury also…so because of this the visits gradually become low.” -FLW supervisor, low-performing block
− Inadequatetechnologyinfrastructure	Barrier: Lack of computers, mobile phones, and manpower to report data on time	“Sometimes reports don't come on time because FLW supervisors don't have phones, so they have to deliver the data in person…they don't have a computer either so they can't upload the data anywhere, nor has any software come up for this purpose.” - Block staff, low-performing block“Main thing is that I don't have the support or manpower to do [data] feeding…I don't have a computer…and if I did, I don't have anyone to do the computer work. We do the data feeding here and there…We go to the shop and ask them to enter the data [on the computer]. Sometimes they do it through mobile.” - Block staff, high-performing block
Use of data		
+ Collaboration	Facilitator: Staff from different agencies sharing and reviewing each other's data sources	“If I have some weakness or MOIC has weakness, we share data like about any pregnant women aged 15–49 and if he gets to know he shares with us and we share with him…[we work] together with synergy like ‘here sir, you need to focus on this point in the meeting.” - Block staff, high-performing block
+ Review meetings	Facilitator: Monthly review meetings between block staff, supervisors, and FLWs	“When ASHAs come to cluster meetings…we put their checklist in front of them and review it together…a formal meeting is sometimes not necessary because people come every day. Sometimes we take 5 to 10 minutes to review with them.” - Block staff, high-performing block“Here we have an HMIS validation committee where [several block staff], and myself are there. Every month we meet…when the data is given by the ANMs, we meet together to discuss its quality.” - Block staff, low-performing block
− Human resourcegap	Barrier: Low staffing decreased the ability for FLW supervisors to use and analyze the supportive supervision checklist for improving FLW performance in counseling	“There are centres where beneficiary said that ASHA used to come and give vaccines but never share any information with us like we have to go for institutional delivery, initiation of breastfeeding within an hour of delivery etc. Workers are aware and get information here but not bother to tell beneficiaries. So I need someone to tell me all these things so we will counsel our workers separately.” - Block staff, high-performing block
− ConflictsbetweenFLWs	Barrier: Lack of cooperation and conflicts on job responsibilities	“During VHND, ASHA and AWW have to work together and have same role of calling people…in some areas, AWW will say that this is not her job, it's for ASHA to do…ASHA will say that working as a team is not my responsibility.” - Block staff, low-performing block “In front of our district magistrate it has shown the percentage of ASHAs present and 7% presence of AWW…sometimes it happens that there is no synergy between the workers.” - Block staff, high-performing block
− Political system	Barrier: Political hierarchy structure and empowerment to make changes	“Any government program is done by government's work structure and according to their mechanism we have to work on. We can't do any changes on them. We have made our system work according to the program or if we get any problem then we try to solve it at our level.” - Block staff, high-performing block “If I was at a higher level, I would be at the policy level trying to make policy changes. Not here doing implementation work with ASHAs.” - Block staff, low-performing block

1 Data source: in-depth interviews with block staff and FLW supervisors. “+” = Facilitator, “−” = Barrier. ASHA, accredited social health activist; ANM, auxiliary nurse midwife; AWW, Anganwadi worker; CHC, community health center; FLW, frontline worker; HMIS, Health Management Information System; MOIC, medical officer in charge; UP, Uttar Pradesh; VHND, Village Health and Nutrition Day.

#### Facilitators for data collection

The 2 primary facilitators for data collection that emerged from the in-depth interviews were block ranking and collaboration. Collaboration was a cross-cutting theme and influenced perceptions on ease of data collection as well as data use.

##### Block ranking

Block staff in the Kanpur-Dehat district described the importance of timely data collection in order to ensure the block's high ranking. If data were not collected regularly or if numbers declined, it may result in a drop in rank for their district and block. Thus, awareness of the importance of data to inform block rank was a motivating factor for data collection.

##### Collaboration

In 1 high-performing block, there was extensive collaboration between block-level staff to overcome logistical barriers that negatively impacted data collection. When issues arose, participants described holding meetings to find solutions together (such as delegating responsibility to other FLWs or having supervisors reach out to absent staff). In addition, 1 solution that helped reduce attendance issues was the creation of a micro-plan between the 2 ministries that implement nutrition and health programs in India. This collaboration was critical to coordinate activities and dates to avoid conflicts and ensure attendance of FLWs across the 2 main government programs.

#### Barriers to data collection

The 3 primary barriers to data collection that emerged from the data were educational level of FLWs, inadequate technology infrastructure, and human resource gaps. Human resource gaps were a cross-cutting theme and influenced perceptions on ease of data collection, data quality, as well as data use.

##### FLW educational level

Suboptimal educational level of auxiliary nurse midwives (ANMs) was cited as a reason for issues with data collection because of their lack of familiarity with technical data. Some participants commented that, although the project has prioritized building the capacity of ANMs in data collection, the training sessions were unsuccessful due to the low educational level of the FLWs and their ability to retain information.

##### Human resource gaps

Human resource gaps including both ANM vacancies and lack of staffing in ICDS was another key challenge for data collection. Without ANMs, the bulk of data collection and reporting was not considered possible by respondents. In the situation of a vacancy, an ANM from a nearby subcenter was requested to collect the data and administer the services at the vacant subcenter, in addition to managing these same responsibilities at her own subcenter. FLW supervisors within the vacant subcenter or from nearby subcenters would also be recruited to assist with data collection and reporting if the ANM was overburdened. Concerns on inadequate staffing were also discussed for ICDS offices that had no data operator on staff and few FLW supervisors. High workload due to staff shortage was reported in low-performing blocks, causing time for supervisory visits to be sacrificed in order to fulfill administrative tasks.

##### Inadequate technology infrastructure

A lack of access to technology was described as a barrier to communication between staff members and timely reporting of data. For example, an FLW supervisor commented that it was easier when they used paper but now that they use mobile phones to enter checklist data she has not done it in over a month due to lack of an internet connection. Likewise, many staff members commented on having backlogs of data to share due to connectivity issues. Some staff commented that the use of technology increased workload, as they would enter data both on hard copy and electronically due to concerns about being able to upload data. Another concern was lack of computers or staff who knew how to use computers to enter and manage data. Both high- and low-performing blocks lacked adequate technology (computers, phones) and staff support to complete data-reporting tasks.

#### Facilitators of data use

The collaboration and review meetings were important factors that facilitated data use for supply chain management and for ranking and evaluating block performance in this project.

##### Collaboration

Collaboration between staff in different government departments [medical officer in charge (MOIC) and CDPO] facilitated the use of data for decision making. The transparent exchange of data between staff members allowed them to clarify future areas for improvement and points for discussion in each department's monthly review meetings. Similarities in indicators across agencies allowed for cross-comparison of results. Block-level staff discussed that any imbalances were detected in the cross-check and could spur a plan of action. Sharing of data across agencies helped ensure a common understanding of the situation, discussion of supply chain management, and identification of target areas for improvement in block performance.

##### Review meetings

Review meetings took place once a month, during which block staff, supervisors, and FLWs met to review data and discuss areas for improvement. For example, MOICs reported how formal and informal review meetings allowed them to provide feedback to FLWs based on supervision checklist data. In most of the blocks, a separate review meeting was organized among the block staff to review the HMIS data in particular, which facilitated its use.

#### Barriers to data use

Human resource gaps, conflicts between FLWs, and the overall political system limited real-time data-driven decision making to improve program implementation.

##### Human resource gaps

Staff vacancies complicated not only data collection but also analysis and use of data to make changes in program implementation. For example, the supportive supervision checklist was reported as a valuable tool to track the performance of FLWs and allow for refresher training on problem areas. However, the low number of supervisors on staff decreased the capacity for FLW supervision and thus the extent to which data could be used to develop targeted solutions for FLWs who demonstrate weaker performance. This was a primary concern across both high- and low-performing blocks.

##### Conflicts between FLWs

In order to implement changes based on the data, block staff described that there must be cooperation between FLWs. In both high- and low-performing blocks, conflicts between FLWs had disrupted the workflow of maternal health service delivery. Lack of job role clarity and collaboration resulted in conflicts among FLWs on whose responsibility it was to act on the data.

##### Political system

When block staff were asked about the actions they are able to take based on the data, a common theme that emerged was the low feasibility of making changes. Staff from both high- and low-performing blocks described that the maternal nutrition services are implemented according to the work structure that the Government of India mandates, leaving little room for changes in data-reporting standards, funding, and recruitment of staff. Block-level staff reported having a limited scope of work and ability to make changes to program operations.

### Quantitative data

Findings from surveys with 103 supervisors showed that the majority of FLW supervisors reported reviewing data on pregnant women (95%), with ∼60% reporting discussing data in FLW meetings (**Table 2**). Approximately one-third of supervisors reported using the data for IFA and calcium-supplement supply chain management. While 50% of FLWs reported using data to identify gaps/areas of improvement, only 23% reported using the data for decision making on areas of improvement for block performance. FLW supervisors reported several challenges in using data on maternal nutrition interventions, including the following: difficulty in understanding data (16%), not feeling confident in data quality (15%), lack of time for interpreting and discussing data (10%), and lack of availability of data (7%).

**TABLE 2:** Data use and challenges among FLW supervisors^1^

	Percentage
Review data on pregnant women	95.1
Use of data	
Data discussed in AAA meetings^2^	58.3
Data discussed in sector/cluster review meeting	61.2
Data used to monitor stock of IFA and/or calcium supplements	32
Data used to identify areas for improvement and gaps	49.5
Data used for decision making on areas for improvement	23.3
Challenges in using data	
Data are difficult to understand	15.5
Do not feel data are accurate/problems in data quality	14.6
Do not feel use of data is important	4.9
Lack of time for interpreting/discussing data	9.7
Data are not available for review/use	6.8
No problem faced	57.3

1 *n *= 103. ASHA, accredited social health activist; ANM, auxiliary nurse midwife; AWW, Anganwadi worker; FLW, frontline worker; IFA, iron and folic acid.

2 Monthly AAA (“triple A”) meetings bring together the ASHAs, AWWs, and ANMs of each block.

For the MN block card, 58% of FLW supervisors had heard of it and 46% reported using it during meetings (**Table 3**). However, only 33% of supervisors reported using the MN block card to identify areas where intervention coverage or service delivery was low, 18% reported using it to prioritize areas of improvement, and 8% of supervisors reported using the data to decide on next steps to improve implementation. Likewise, there was high awareness of the supportive supervision checklist (86% had ever heard of it); >50% reported using it to identify areas of high- and low-quality counseling. However, only 16% reported using the checklist to decide on next steps to improve the quality of maternal nutrition counseling.

**TABLE 3:** Use of MN block cards and supportive supervision checklist data among FLW supervisors^1^

	Percentage
Heard of MN block card	58.3
MN block card used during sector/cluster review meetings	45.6
Use of MN block card data	
Discuss status of MN indicators in the block for each month	27.2
Identify areas where block has progressed on MN	33
Identify areas where level of coverage/service delivery is low	33
Prioritize areas for improvement	17.5
Decide on next steps to achieve improvements in identified areas	7.8
Heard of supportive supervision checklist	86.4
Supportive supervision checklist used during sector/cluster review	79.6
Use of supportive supervision checklist data	
Quality of counseling on MN topics discussed	40.8
Identify areas/topics with high quality of counselling by FLWs	50.5
Identify topics where quality of counseling is low	54.4
Prioritize topics for improving quality of counseling on MN	38.8
Decide next steps to improve quality of MN counseling in block	15.9

1 *n *= 103. FLW, frontline worker; MN, Maternal Nutrition.

## Discussion

This mixed-methods study provides an in-depth understanding of data collection and use in the context of a large, ongoing, maternal nutrition intervention in Uttar Pradesh, India. Overall, there were intensive data-collection efforts and high staff awareness of data sources. Data were used for supply chain management and to monitor and improve block performance, including how data were used to provide supportive supervision and monitor quality of counseling and service provision in communities. However, the reported use of data to inform decision making and take corrective action was low in this setting, with only 23% of FLW supervisors reporting using data for decision making on areas of improvement. Our study provides a valuable case example of the real challenges as well as opportunities that programs face as they aim to scale up data-driven accountability efforts called for in the recent 2021 Lancet Nutrition Series (6).

The pathway from data collection to data use was influenced by multiple facilitators and barriers, some of which have been addressed in previous literature regarding use of monitoring data (11, 13). Challenges in technology infrastructure were key barriers to data collection. In a systematic review of mobile health interventions, mobile technology tools were shown to help community health workers improve care, communication between workers, and program-monitoring data collection (28). However, the availability of phones is critical to achieve these objectives and must be coupled with technical support to health workers, adequate mobile network availability, and data security to ensure its sustainability (29). In addition, in our study, staff vacancies created challenges for timely and accurate data collection and utilization. While vacancies in nurse-midwife positions have not been previously cited as a barrier to data collection, staffing vacancies have been linked to poor maternal and newborn health delivery in other low- and middle-income settings (30, 31).

Conflicts between FLWs and low feasibility of decision making due to political hierarchies inhibited use of data to improve the delivery of maternal nutrition services. The lack of autonomy to take corrective action based on the data reported in in-depth interviews was likewise supported by the quantitative survey, which reported low levels of use of data for decision making. Likewise, prior studies have documented how FLWs are negatively affected by the disconnect between district and blocks because they may not be given a voice to explain the reality of health care delivery in the field, which can influence the success of implementing top-down changes (32). Further research is needed to understand how political and social system barriers and lack of autonomy for decision making may impact on work satisfaction and motivation among block-level staff. While caste was not discussed among participants in our study, given the known social dynamics in this context, further research may be merited to understand the role of social structure in collaboration and program implementation (33, 34).

Collaboration and monthly review meetings between FLWs, supervisors, and block staff were key facilitators for data use in this study. Cooperation across health sectors and between districts and blocks is needed for scaling maternal and child health and nutrition interventions. For example, in prior research, ICDS and National Rural Health Mission in Odisha, India, collaborated often to develop guidelines and review programs due to shared motivation and leadership for coordination, which have helped the state close the gap in maternal and child health outcomes between disadvantaged groups and the state average (35). In addition, in our study, block ranking emerged as a key facilitator for data collection. This parallels experience with the baby-friendly hospital ranking and how this has facilitated the collection of breastfeeding data in hospitals (36, 37).

Strengths of our study include the use of mixed-methods that allowed for detailed insight on barriers to and facilitators of data collection and use. The quantitative endline survey was valuable for supporting and complementing the findings from the in-depth qualitative assessment. Additionally, both independent data sources were used to corroborate key messages and allowed for data triangulation on specific findings supporting the quality of the research. For example, results from surveys showed that ANM vacancies were a key challenge for data collection, and results from in-depth interviews revealed that lack of staffing was a barrier to data use. Finally, our study strategically sampled high- and low-performing blocks in order to help ensure diverse perspectives and experiences with data use and program implementation.

There are also important limitations of our study. While most interviews were conducted in private and comfortable settings, this was not always feasible. In blocks with heavy patient traffic, interviews were sometimes interrupted with other staff entering the office space, which may have influenced response bias and inclination to share sensitive or negative information. Given the larger number of interviews completed in private settings than those completed in heavy-traffic settings, we consider the sample and study results to have captured sensitive or negative information. FLW supervisors were selected based on their response to a phone call and willingness to be interviewed. Those who were not interviewed may have had a different perspective on the topics covered in the interview guide. Selection bias was minimized by sample selection methods that included a random selection of which FLWs to approach first in each block, which allowed obtaining a final diverse sample from high- and low-performing blocks at all levels (managers, medical officers, and FLWs) and ensuring to capture the full spectrum of opinions and experiences on data-use barriers and facilitators.

In conclusion, the use of data for decision making is critical for supporting intervention planning and providing targeted supervision and support for FLWs. Our study provides novel insight into the key barriers to and facilitators of data collection and use in the context of large maternal nutrition interventions and has important implications for program implementation. Collaboration between agencies through regular intersectoral meetings and the transparent exchange of data facilitated data-collection and utilization efforts. Block-level review meetings provided a valuable platform for data-quality review and decision making. Further investment may be required to build the capacity of block-level staff to implement contextually relevant nutrition programs and to empower them to make data-driven decisions to improve program performance. Addressing structural barriers, such as staff vacancies, lack of technology infrastructure, and training, is required to facilitate data collection and data use and thus improve the implementation of maternal nutrition interventions. Future research on the importance and impact of real-time data use for guiding decision making across different contexts is needed to help prioritize and allocate resources to scale-up global data-driven accountability efforts.

## Supplementary Material

nzab081_Supplemental_File

## Notes

Supported by the Bill & Melinda Gates Foundation (through Alive & Thrive, managed by FHI Solutions, grant number OPP50838) and Emory University.

Author disclosures: The authors report no conflicts of interest.

Supplemental Figure 1 and Supplemental Material are available from the “Supplementary data” link in the online posting of the article and from the same link in the online table of contents at https://academic.oup.com/cdn/.

Abbreviations used: A&T, Alive & Thrive; ANM, auxiliary nurse midwife; CDPO, child development program officer; FLW, frontline worker; HMIS, Health Management Information System; ICDS, Integrated Child Development Services; IFA, iron and folic acid; IFPRI, International Food Policy Research Institute; MN, Maternal Nutrition (block card); MOIC, medical officer in charge.
