# Supplementary material for: Understanding Implementation and Improving Nutrition Interventions: Barriers and Facilitators of Using Data Strategically to Inform the Implementation of Maternal Nutrition in Uttar Pradesh, India
Source: Curr Dev Nutr. 2021 Jun 2;5(6):nzab081. doi: 10.1093/cdn/nzab081 (PMC8242137; doi:10.1093/cdn/nzab081)
Supplement: nzab081_Supplemental_File [file nzab081_supplemental_file.docx]

**Understanding implementation and improving nutrition interventions: Barriers and facilitators of using data strategically to inform the implementation of maternal nutrition in Uttar Pradesh, India. Young, MF et. al.,**

**Online Supplementary Material**


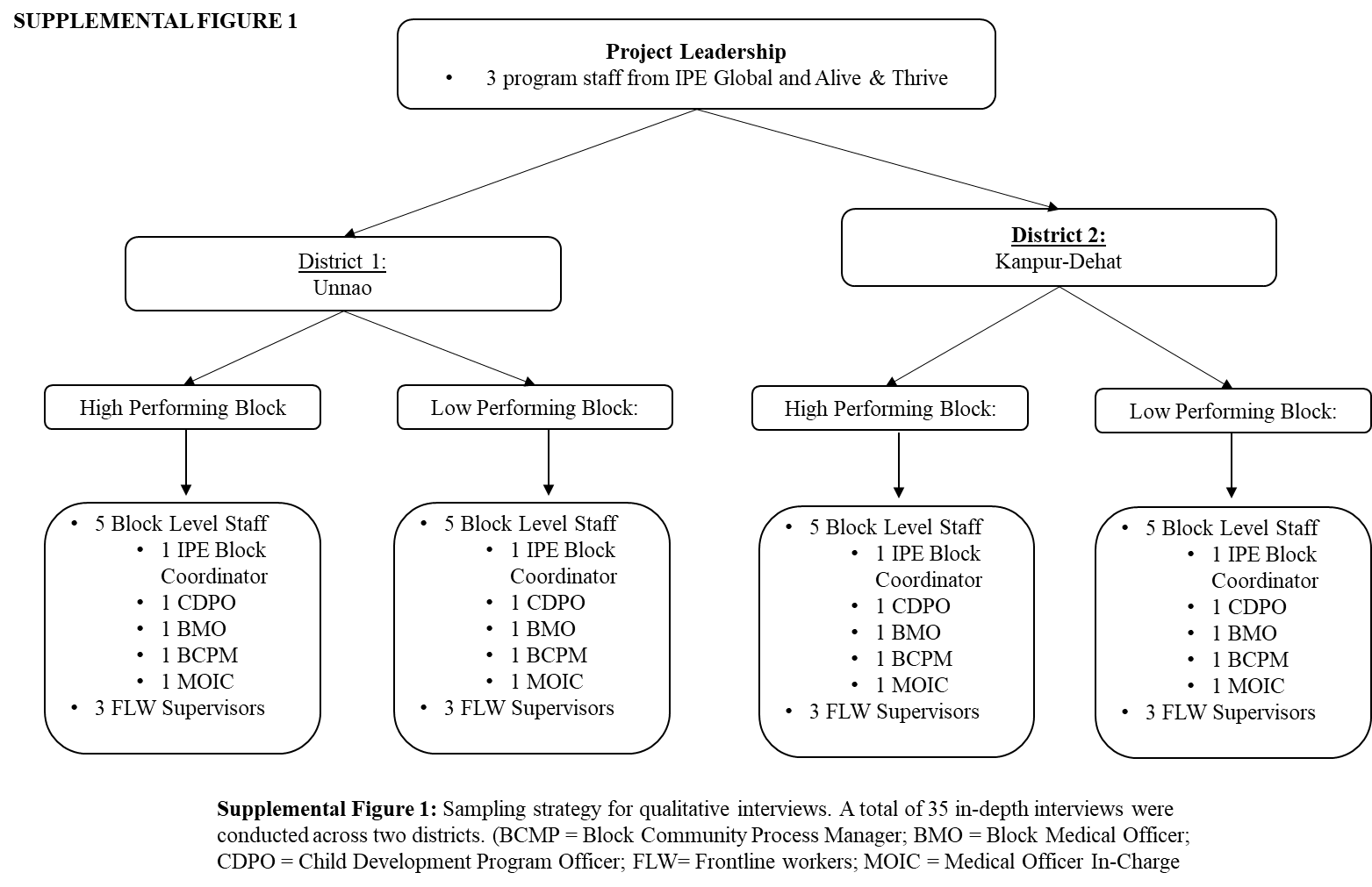


**ONLINE SUPPLEMENTAL MATERIALS: QUALITATIVE TOOLS**

## Alive & Thrive In-Depth Interview (IDI) Guide

Warm-Up:

1. Tell us about your role as xxx. What do you specifically do for the maternal nutrition program?
   1. *Responsibilities?*
   2. *What does your daily work involve?*
   3. *How does it fit in with overall plans for the program?*
2. What has your professional experience been in implementing the maternal nutrition program?
   1. *Positive and negative experiences?*
   2. *How long have you been involved?*

Supply of IFA and Calcium supplements

1. Please describe the steps taken to ensure consistent supply of IFA supplements.
   1. *What is the process for estimating the required supply?*
   2. *Who is involved in monitoring stock of supplements?*
   3. *What’s the process of delivery to the households?*
   4. *How often is stock checked at health sub-centers?*
   5. *Storage and inventory management?*
   6. *Record-keeping of stock transactions (receipt of supplements, consumption, adjustments, losses, any other logistical issues)?*
2. What have been the challenges of maintaining adequate stock of IFA supplements?
   1. *Obtaining supplements?*
   2. *Storage of supplements?*
   3. *Feedback on top-down distribution of supplements?*
3. What do you think can be done to improve the supply of IFA supplements?
   1. *Thoughts on streamlining the process?*
   2. *Suggestions for top-down distribution from manufacturers?*
   3. *Suggestions on horizontal allocation of supplements to PHC/CHC and HSCs?*
4. Until now, we have talked about maintaining supply of IFA supplements. How does this compare to the supply of calcium?
   1. *What is the process for estimating the required supply?*
   2. *Who is involved in monitoring stock of calcium supplements?*
   3. *What’s the process of delivery to the households?*
   4. *How often is stock checked at health sub-centers?*
   5. *Storage and inventory management?*
   6. *Record-keeping of stock transactions (receipt of supplements, consumption, adjustments, losses, any other logistical issues)?*
5. What have been the challenges of maintaining adequate stock of calcium supplements?
   1. *Obtaining supplements?*
   2. *Storage of supplements?*
   3. *Feedback on top-down distribution of supplements?*
6. What do you think can be done to improve the supply of calcium supplements?
   1. *Thoughts on streamlining the process?*
   2. *Suggestions for top-down distribution from manufacturers?*
   3. *Suggestions on horizontal allocation of supplements to PHC/CHC and HSCs?*
7. What data is being collected on IFA and calcium supplementation?
   1. *Stock of supplements?*
   2. *Whether supplements were delivered to mothers?*
   3. *Coverage (amount of households, ANC centers, and blocks that receive adequate amount of supplements)?*
   4. *Consumption/intake?*
   5. *How frequently is all of this monitored?*
   6. *Who is collecting monitoring data?*
8. How is this data being used for decision making and managing operations?
   1. *Storage management?*
   2. *Inventory management?*
   3. *Supply chain decisions upstream?*

Counseling on recommended maternal nutrition services

1. How is the program influencing counseling services delivered to pregnant women?
   1. *Counseling on dietary diversity and quantity of food intake?*
   2. *Consumption of IFA and calcium supplements?*
2. What have been some challenges in strengthening the counseling services component of the program?
   1. *Keeping FLWs motivated?*
   2. *Training of FLWs?*
   3. *Motivation of supervisory staff?*
   4. *Adherence to counseling messages?*
   5. *How have you been addressing these challenges?*
3. What data is being collected on counseling services?
   1. *At household?*
   2. *At VHNDs?*
   3. *At anganwadi centers (AWCs)?*
   4. *How is the data being collected?*
   5. *How is it used?*
   6. *Effect on program operations?*

Strategic use of data:

1. Besides data on supplements, counseling services, and ANC, what data is A&T monitoring for program implementation?
   1. *Quarterly household surveys? Indicators?*
   2. *Supportive supervision checklist? Indicators?*
   3. *Program activity sheets? Indicators?*
   4. *Sources of these data?*
   5. *Other key indicators you would recommend adding to any of the monitoring data methods?*
   6. *How would these be helpful to the program?*
2. How do you access the program monitoring data?
   1. *Timeliness of accessing data? (how soon after collected do you see?)*
   2. *Challenges in accessing, interpreting?*
   3. *Thoughts on quality of data? How do you define quality?*
   4. *Advantages of collecting monitoring data?*
   5. *Improvements you recommend?*
3. How is the program monitoring data currently used?
   1. *Sharing with other team members?*
   2. *Decisions to be made from the data?*
   3. *Changes in the training of FLWs and supervisors?*
4. How do you use the government monitoring (HMIS) data?
   1. *Preparation of MN block cards?*
   2. *Challenges in using HMIS data for block cards?*
   3. *Perceptions on quality of data?*
   4. *Advantages of using this data?*
   5. *Improvements you recommend?*
5. How do you think the MN data dashboard efforts will be scaled-up to the current ANC platform in the health system?
   1. *Feasibility?*
6. What are the challenges of scaling up A&T’s program initiatives to the entire district or state?
   1. *Opinions of state and district leadership?*
   2. *Staffing capacity?*
   3. *Funding capacity?*
   4. *Internal relationships between politicians?*
7. What are the internal program activity targets you discuss in the quarterly review meetings with IPEG?
   1. *Current targets?*
   2. *How are targets set?*
   3. *How do you monitor targets?*
   4. *Are you meeting the targets?*
   5. *What targets are not being met and how can they be met?*
   6. *Is program monitoring data discussed here? If so, how is it being used?*
   7. *Challenges of conducting review meetings?*

Closing:

1. What recommendations do you have to improve the data collection and use of program monitoring data?
2. Is there anything else you would like to share?

Thank you very much for your time and valuable insights!

## Program Staff IDI Guide

Warm-Up:

1. Can you describe your role in the implementation of Maternal Nutrition program?
   1. *Responsibilities?*
   2. *What does your daily work involve?*
   3. *How does it fit in with overall plans for the program?*
2. What has your experience been in implementing the maternal nutrition program?
   1. *Positive and negative experiences?*
   2. *How long have you been involved?*

Capacity Development/Training

1. Can you describe how the field team (district manager, block coordinators, and field monitors) was oriented?
   1. *Components of the orientation?*
   2. *When were they oriented?*
   3. *Length of orientation?*
2. Can you describe how the supervisors are trained on supporting the FLWs?
   1. *Training components? (Completing supportive supervision checklists? Contact points with pregnant women? Record keeping on MCTS? Supplies management? Accompanying FLWs during counseling sessions?)*
   2. *When were they trained?*
   3. *Length of training?*
3. How are the FLWs trained on knowledge of maternal nutrition and counseling pregnant women?
   1. *Training components? (Counseling on IFA intake? Counseling on Calcium intake? Weight monitoring? Counseling on weight gain? Counseling on diet diversity at home visits? Seeking feedback from pregnant women?)*
   2. *When were they oriented?*
   3. *Length of orientation?*
   4. *Is refresher training conducted at monthly meetings? What do you review in these trainings?*
4. How do you think the trainings have impacted the knowledge and skills of government supervisors and block-level managers?
5. How do you think the trainings have impacted the knowledge and skills of FLWs?
6. What have been the challenges in training the government supervisors and block-level managers?
   1. *Not enough time?*
   2. *Drop-outs?*
   3. *Encouraging use of SS checklists?*
7. What have been the challenges in training the FLWs?
   1. *Not enough time?*
   2. *Drop-outs?*
   3. *Attendance at monthly meetings for refresher trainings?*
   4. *Engagement from supervisors?*

Service Delivery & Community Mobilization

1. How is IPE Global strengthening the delivery of maternal nutrition services?
   1. *At PMSMAs?*
   2. *At home visits?*
   3. *At VHNDs?*
2. What have been the challenges to delivering these services in each of these settings?
   1. *PMSMAs?*
   2. *Home visits?*
   3. *VHNDs?*
3. How is IPE Global engaging the community in supporting maternal nutrition practices (regular antenatal care, tracking weight gain of mother, dietary diversity, and consuming IFA and calcium supplements)?
   1. *Participation of husbands/men at Ratri Chaupals? Mother-in-laws? Other family members?*
   2. *Engaging local religious leaders and village elders?*
   3. *Involving local health care providers?*
   4. *Is there documentation of participants?*
4. What challenges have you faced in conducting the Ratri Chaupals, if any?
   1. *Participation from husbands? Other family members?*
   2. *Participation from any community members?*
   3. *Attendance?*
   4. *Facilitating the RCs?*
   5. *Showing the videos on maternal nutrition?*

Strategic use of data:

1. What types of data is IPE Global collecting and/or using from village, block, and districts?
   1. *MN block cards?*
   2. *Performance of FLWs (SS checklists at VHND, home visit, and RCs)?*
   3. *Quarterly household survey?*
   4. *VHSND and RC attendance?*
   5. *Sub-center operations?*
   6. *Support from supervisors?*
2. Please describe the supportive supervision checklists.
   1. *Do you have these checklists?*
   2. *Walk through process of how they’re used in the field? (where? by who? when?)*
   3. *Frequency of checklist completion?*
   4. *Frequency of sharing checklist data and with who?*
3. How are the data from the SS checklists used?
   1. *Use in review meetings?*
   2. *Use for program operations?*
   3. *Feedback given to supervisors? FLWs?*
4. How do you prepare the program monitoring data?
   1. *Timeliness of accessing data? (how soon after collected do you see?)*
   2. *Challenges in accessing, interpreting?*
   3. *Perceived quality of data? How do you define quality?*
   4. *Advantages of collecting monitoring data?*
   5. *Improvements you recommend?*
5. How is the program monitoring data currently used?
   1. *Sharing with other team members?*
   2. *Decisions to be made from the data?*
   3. *Changes in the training of FLWs and supervisors?*

19. How do you use the government monitoring (HMIS) data?

- 1. *Preparation of MN block cards?*
  2. *Challenges in using HMIS data for block cards?*
  3. *Perceptions on quality of data?*
  4. *Advantages of using this data?*
  5. *Improvements you recommend?*

1. What has been your experience in developing MN report cards?
   1. *Access to HMIS data?*
   2. *Quality of HMIS data?*
   3. *Identifying elements for the card?*
   4. *How are the percentages calculated to put into the MN block cards?*
2. How are MN report cards currently used?
   1. *Challenges in using cards?*
   2. *Orienting government staff to use cards?*
   3. *Discussed at monthly meetings?*
   4. *Decisions based on card?*
3. Please describe the weekly review meetings and points/targets discussed.
   1. *Who conducts them, where, and how frequently?*
   2. *Targets/points discussed?*
   3. *How are targets set?*
   4. *How do you monitor targets?*
   5. *Are you meeting the targets?*
   6. *Actions taken when targets are not met?*
   7. *How can they be met?*

1. Please describe the monthly review meetings and use of monthly program targets?
   1. *Who conducts them, where, and how frequently?*
   2. *Targets/points discussed?*
   3. *How are targets set?*
   4. *How do you monitor targets?*
   5. *Are you meeting the targets?*
   6. *Actions taken when targets are not met?*
   7. *How can they be met?*

Closing:

1. Please comment on the sustainability of IPE’s systems-strengthening activities.
   1. *How are independent VHNDs with MN counseling possible? What makes them successful and why?*
   2. *How are independent RCs with MN counseling possible? What makes them successful and why?*
2. What are the challenges of scaling up A&T’s program initiatives to the entire district or state?
   1. *Opinions of state and district leadership?*
   2. *Staffing capacity?*
   3. *Funding capacity?*
   4. *Internal relationships between politicians?*
3. What recommendations do you have to improve the data collection and use of monitoring data?
   1. *For A&T program monitoring data?*
   2. *For government/HMIS data?*

Is there anything else you would like to share?

Thank you so much for you time and valuable insights!

## Block Coordinator IDI Guide

1. Can you describe your role in the implementation of Maternal Nutrition program?
   1. *Responsibilities?*
   2. *What does your daily work involve?*
   3. *How does it fit in with overall plans for the program?*
2. What has your experience been in implementing the maternal nutrition program?
   1. *Positive and negative experiences?*
   2. *How long have you been involved?*

Program Implementation

1. Please describe your process for activating a sub-center.
   1. *Village meetings with AWWs, ANMs, and ASHAs?*
   2. *VHSNC meetings?*
   3. *What leaders/influencers do you engage?*
   4. *Key messages conveyed?*
2. How have you been strengthening the delivery of maternal nutrition services at VHNDs?
   1. *Ensuring adequate supplies of IFA and Ca supplements?*
   2. *Supporting weight measurement of mothers?*
   3. *Planning meetings with BCPM and ANM?*
   4. *Documentation of VHND attendance and activities?*
3. What has been your experience in strengthening the services at VHNDs?
   1. *Things that have been successful?*
   2. *Challenges faced?*
4. What has been your experience in facilitating Ratri Chaupals?
   1. *Engaging husbands? Other family members?*
   2. *Challenges?*
5. How have you been strengthening the interpersonal counseling skills of FLWs?
   1. *Are you doing joint mentoring sessions with supervisors?*
   2. *Observations from home visits? From VHNDs? From group counseling at VHND/Mamta Diwas/Godbharai events?*
   3. *Feedback given to FLWs? To supervisors? Any examples?*

Performance Improvement

1. How are you monitoring the performance of FLW supervisors, if at all?
   1. *Collecting any data?*
   2. *Using program activity sheets? HH surveys? SS checklists? How?*
   3. *How do you define quality of work? Performance?*
   4. *Discussing in monthly review meetings?*
2. How are you monitoring the performance of FLWs, if at all?
   1. *Collecting any data?*
   2. *Using program activity sheets? HH surveys? SS checklists?*
   3. *Discussing in monthly review meetings?*

Closing

1. What recommendations do you have to improve the implementation of the maternal nutrition program?
   1. *Supply chain management?*
   2. *Training of supervisors and FLWs?*
   3. *Supervision of FLW supervisors and FLWs?*

Is there anything else you would like to share?

Thank you so much for you time and valuable insights!

## Block-Level Program Staff IDI Guide

Warm-Up:

1. What role do you play in the implementation of the maternal nutrition services in this block?
   1. *What are your responsibilities and contributions to the program?*
   2. *What does your daily work involve?*
   3. *How is your role different from other government block staff?*
2. What has been your experience in strengthening and prioritizing maternal nutrition service delivery through the current ANC platform?
   1. *How long have you been involved?*
   2. *Please describe your positive and negative experiences.*

Delivery and Quality of the maternal nutrition services:

1. How are the current maternal nutrition services being delivered in the block?
   1. *What services are delivered at health facilities such as PHC or CHC?*
   2. *What services are delivered at the health sub-center?*
   3. *What services are delivered at the VHNDs?*
   4. *What services are delivered at the household?*
   5. *Who’s involved in delivery?*
2. How is the delivery of maternal nutrition services being monitored?
   1. *What’s being monitored? In what settings are you monitoring these services?*
   2. *Who’s monitoring the services?*
3. Based on what you’ve observed/heard, what changes have been seen in the use and coverage of maternal nutrition services in the last year?
   1. *Have they increased, decreased, or stayed the same?*
   2. *What types of services are being utilized more or less?*
   3. *Is data being monitored on these services?*
4. What has been your experience in working with IPE Global staff in delivering maternal nutrition services in your area?
   1. *Can you describe your orientation to the IPE program?*
   2. *Do you think IPE Global’s work has been helpful? Why or why not?*
   3. *What have been IPE Global contributions to delivery of maternal nutrition services?*
   4. *What challenges have you encountered when working on IPE Global’s initiatives?*
5. What have been the challenges of implementing the maternal nutrition program?
   1. *Any difficulty with accessing or understanding monitoring data?*
   2. *Any challenges with compliance from mother and family members to maternal nutrition messages delivered during counseling*
   3. *Any challenges with FLW performance and motivation?*
   4. *Any challenges with the availability of IFA and calcium?*
   5. *Any challenges with communicating with program staff members or field workers?*

Supportive supervision of government program staff:

**FOR HEO + BPM (ANM Supervisor):**

1. Can you describe your experience supervising the ANMs?
   1. *How many ANMs do you supervise?*
   2. *What activities by the ANMs do you supervise?*
   3. *Can you please comment on the ANMs’ knowledge of diet and nutrition during pregnancy and post-partum?*
   4. *Can you please comment on the ANMs’ knowledge of breastfeeding practices?*
2. Can you describe a typical supervision visit to ANMs in your area?
3. *Where do visits take place?*
4. *What activities do you perform during your supervision visits?*
5. *Do you provide feedback to the ANMs? What feedback do you provide?*
6. *How are the ANMs using your feedback?*
7. What were some difficulties, if any, that you encounter when trying to supervise the ANMs?

**FOR ALL BLOCK GOVERNMENT STAFF:**

1. Can you describe your experience with conducting your supportive supervision visits to households and VHNDs?
   1. *What activities do you perform during your supervision visits?*
   2. *Have you been able to complete all these visits? Why or why not?*
   3. *What challenges have you faced, if any, to complete these visits?*
2. Please describe your experience with the supportive supervision checklists.
   1. *What indicators are you monitoring? How frequently?*
   2. *How is data collected?*
   3. *What have been some challenges to data collection?*
   4. *Who is this shared with?*
   5. *Have there been any challenges with sharing the checklist data?*
3. How do you use the supportive supervision checklists?
   1. *Are you giving any feedback to FLWs based on the checklist data? If not, why?*
   2. *Are you discussing the data in any meetings, such as cluster or sector meetings?*
   3. *How does the checklist data influence program operations?*
   4. *Are the supportive supervision checklists helpful, in your opinion?*

Strategic use of data:

1. What types of data do you have access to?
   1. *Do you have access to HMIS data?*
   2. *Do you have access to IPE program monitoring data? These include supportive supervision checklists, program activity sheets, and quarterly household surveys.*
   3. *What indicators are you monitoring?*
2. What difficulties do you encounter in accessing the data?
   1. *Is the data regularly shared with you? Why or why not?*
3. What data do you use and not use?
   1. *Do you use HMIS data? How?*
   2. *Do you use IPE programing monitoring data? How?*
   3. *Why do you use these data?*
   4. *Why do you not use these data?*
4. How do you use these data?
   1. *What data do you look at?*
   2. *What actions do you typically take based on the data?*
   3. *Are you discussing the data in any meetings, such as cluster or sector meetings?*
   4. *Who do you share the data with, if at all?*
5. Please describe the quality of the data you are accessing.
   1. *Why do you think this?*
   2. *Are you getting data from all the health sub-centers in your block?*
   3. *Are you missing any data from sub-centers? If so, why?*
   4. *Are you missing any data on certain indicators?*
   5. *How frequently do you get these data?*
6. What are the challenges of using this data?
   1. *Can you make changes to program operations based on the data? Why or why not?*
   2. *Is more data needed?*
7. What has been your experience with the MN block cards?
   1. *Are you aware of it?*
   2. *Do you play any role in developing these cards? If so, what is your role?*
   3. *What indicators do the block cards contain?*
   4. *How is MN block card data different from the program monitoring data?*
8. How do you use the MN block cards?
   1. *What actions do you typically take based on the data?*
   2. *Are you using them to review performance of supervisors or FLWs? If so, how?*
   3. *What indicators are you looking at?*
   4. *Are you discussing them in sector or cluster level meetings? If so, how?*
   5. *Who do you share the MN block card data with, if at all?*
9. Based on your personal experience, how valuable is collecting data for program implementation?
   1. *In your opinion, should program monitoring continue? Why or why not?*
   2. *What insights have you gained about maternal nutrition services by using the data?*

Closing:

1. How do you think IPE’s program interventions can be strengthened?
   1. *Do you think Ratri Chaupals will be sustainable in the future? Why or why not?*
   2. *Do you think the checklists introduced by IPE Global will integrated into the current government health system? Why or why not?*
   3. *What is the potential to scale-up these interventions to other districts and the entire state?*
2. What recommendations do you have to improve the data collection and use of monitoring data?
   1. *For IPE Global’s program monitoring data?*
   2. *For government/HMIS data?*

Is there anything else you would like to share?

Thank you very much for your time and valuable insights!

## Front Line Worker (FLW) Supervisor IDI Guide

| Designation of supervisor: |  |
| --- | --- |
| Age: | Village: |
| Years working: | Education: |

Training exposure and availability of materials and supplies

1. Have you received a training on maternal nutrition from IPE Global in the last year?
   1. *When did the training happen?*
2. Can you tell me about your experience with the trainings?
3. *What were the topics discussed at the trainings?*
4. *How did you feel about trainings? Were they useful? Why/why not?*
5. *Is there anything unclear or something you would wish has been covered?*
6. *After the initial training, did you receive any additional refresher training/coaching on maternal nutrition?*

Work

1. Can you describe what your work responsibilities are?
2. What materials and tools do you use to deliver maternal nutrition services?
3. What difficulties have you faced in carrying out the nutrition related services you are responsible for?

Home visits and check-ups/ Interpersonal counseling

**For Lady Supervisor:**

1. Can you describe your experience supervising the AWW?
2. *How many AWWs do you supervise?*
3. *What activities performed by the AWW do you supervise?*
4. *Where do you conduct these supervision visits? (At home? At VHND? At AWC events?)*
5. *Have you been able to complete all these visits? Why or why not?*
6. *Can you please comment on the AWWs knowledge of diet and nutrition during pregnancy and post-partum?*
7. *Can you please comment on the AWWs knowledge of breastfeeding practices?*
8. Can you describe a typical supervision visit to the AWC in your area?
   1. *What activities do you perform during your supervision visits?*
   2. *Do you provide feedback to the AWWs? What feedback do you provide?*
   3. *How are the AWWs using your feedback in their daily work?*
9. What are some difficulties, if any, that you encounter when you supervise the AWWs?

**For ASHA Sangini:**

1. Can you describe your experience supervising the ASHAs?
   1. *How many ASHAs do you supervise?*
   2. *What activities performed by the ASHA do you supervise?*
   3. *Where do you conduct these supervision visits? (At home? At VHND? At CHC/PHC?)*
   4. *Have you been able to complete all these visits? Why or why not?*
   5. *Can you please comment on the ASHAs knowledge of diet and nutrition during pregnancy and post-partum?*
   6. *Can you please comment on the ASHAs knowledge of breastfeeding practices?*
2. Can you describe a typical supervision visit with the ASHAs?
   1. *What activities do you perform during your supervision visits?*
   2. *Do you provide feedback to the ASHAs? What feedback do you provide?*
   3. *How are the ASHAs using your feedback in their daily work?*
3. What are some difficulties, if any, that you encounter when you supervise the ASHAs?

**Continue for ALL Supervisors:**

1. How did you overcome these difficulties?
   1. *What strategies have you implemented to overcome these difficulties?*
   2. *How can these be addressed in the future?*
   3. *Do you mention these difficulties during sector or cluster review meetings?*
2. Please describe how the sector or cluster review meetings are conducted.
   1. *Who leads them?*
   2. *What topics do you discuss?*
   3. *Do you discuss any data at the meetings? If so, what data is discussed?*
   4. *What challenges do you face in conducting these meetings?*
3. How do these meetings compare to the AAA meetings?
   1. *Who leads them?*
   2. *What topics do you discuss?*
   3. *Do you discuss any data at the meetings? If so, what data is discussed?*
   4. *What challenges do you face in conducting these meetings?*
4. What are the steps that need to be taken to improve the delivery of maternal nutrition services?

Community events: VHND

1. What was your experience with organizing VHNDs in the last one year?
2. *Have there been any changes to the way you organized VHND and to the services you provide during VHND?*
3. *What are some challenges in organizing and providing services during VHND?*
4. *Do pregnant women and their families attend? Why? Why not?*
5. *What works well in the VHND?*
6. *How are the current VHNDs helping to strengthen maternal nutrition as compared to previous VHNDs?*
7. *What would you do differently next time you organize a VHND?*

Community events: Ratri Chaupal

1. What was your experience with Ratri Chaupal?
2. *What are some of the challenges with organizing the Ratri Chaupals?*
3. *What worked well in the Ratri Chaupal?*
4. How is the community responding to Ratri Chaupals?
5. *Difficulties for husbands and families of pregnant women to attend?*
6. *Difficulties for other community members to attend?*
7. *Do you feel it will be possible for you and other FLWs to conduct Ratri Chaupals in the future with support from Panchayati Raj Institutions? Why or why not?*
8. *How do you think Ratri Chaupals are helping to strengthen awareness of maternal nutrition in the villages?*
9. *How has this event impacted knowledge of maternal nutrition among community members?*

Strategic use of data

1. What types of data do you have access to?
   1. *Do you have access to HMIS data?*
   2. *Do you have access to IPE Global’s program monitoring data?*
   3. *Do you have access to MN block cards?*
   4. *How is this data used in your daily work?*
   5. *Any challenges to accessing or using the data?*
2. Please describe your experience with the supportive supervision checklists.
   1. *What indicators are you monitoring? How frequently?*
   2. *How is data collected?*
   3. *What have been some challenges to data collection?*
   4. *Who is this shared with?*
   5. *Have there been any challenges with sharing the checklist data?*
3. How do you use the supportive supervision checklists?
   1. *Are you giving any feedback to FLWs based on the checklist data? If not, why?*
   2. *Are you discussing the data in any meetings, such as cluster or sector meetings?*
   3. *How does the checklist data influence program operations?*
   4. *Are the supportive supervision checklists helpful, in your opinion?*

Opinions and perceptions

1. What changes, if any, have you noticed in the maternal nutrition practices of the pregnant women?
2. *Changes in weight gain monitoring?*
3. *Changes in consumption of IFA supplements?*
4. *Changes in consumption of calcium supplement?*
5. *Changes in eating a diverse diet?*
6. *Changes in breastfeeding practices?*
7. What changes, if any, has the maternal nutrition program had on the communities in which you work?
8. *Changes in their knowledge of maternal nutrition practices?*
9. *Changes in their attendance at VHNDs or other community events?*
10. *Changes in the behaviors of local doctors?*
11. According to you, what steps need to be taken to support women and community members in adopting practices?

Closing

1. What recommendations do you have for further efforts in maternal nutrition service delivery?
2. Is there anything else you would like to share?

Thank you very much for your time and valuable insights!

**Acronyms (in alphabetical order):**

BCPM = Block Community Process Manager, HEO = Health Education Officer, BPM = Block Program Manager, CDPO = Child Development Program Officer

**ONLINE SUPPLEMENTAL MATERIALS: QUANTATIVE TOOLS**

Supervisor Questionnaire

International Food Policy Research Institute (IFPRI)

**MODULE: DATA USE AND SECTOR/CLUSTER MEETING**

| **No** | **Question** | **Respond** | **Response code** |
| --- | --- | --- | --- |
| D26a | Do you review/use data on pregnant women and services provided to them? |  | 1. Yes 🡪 D26c 2. No   If no, skip to D26 after asking next question |
| D26b | What are the reasons for not reviewing/using these data?  *(Multiple responses possible)* |  | 1. Do not find it useful to review/use data 2. Did not know about such data 3. Data is not available for review/use 4. Supervisor did not provide instructions 5. Lack of time 6. Not part of job responsibility   95. Other (specify) |
| D26c | According to you, what is the purpose of collecting data related to pregnant women and services provided to them?  *(Multiple responses possible)* |  | 1. To understand situation of health and nutrition in the community 2. To understand status of health and nutrition service delivery 3. To identify areas for improvement in services 4. To decide on actions to improve services and health and nutrition status 5. To monitor status of IFA and calcium stock 6. Other (specify) 7. Don’t know |
| D26d | What types of data do you review/use on pregnant women and services provided to them?    *(Multiple responses possible)*  Prompt: In monthly reporting format under HMIS |  | 1. Data on number of PW registered for ANC 2. Data on number of PW registered in first trimester 3. Number of PW given 180 IFA tablets 4. Number of PW given 360 calcium tablets 5. Number of PW given deworming tablet 6. Number of PW who receive 4 or more ANC check-ups 7. Number of PW tested for haemoglobin during ANC (4 or more times) 8. Number of PW with anemia (Hb <11) 9. Data on stock of IFA supplements 10. Data on stock of calcium supplements 11. Number of households in area 12. Number of reproductive age women 13. Number of mothers with children <6m 14. Number of mothers with children <24m 15. Other (specify) |
| D26e | How often do you review/use these data? |  | 1. Quarterly 2. Every two months 3. Once a month 4. More than once a month   95. Other (specify) |
| D26f | In what ways are these data used?  *(Multiple responses possible)* |  | 1. Data are not used 2. Data discussed in AAA meetings 3. Data discussed in sector/cluster review meeting 4. Data used to monitor stock of IFA and/or calcium supplements   Data used to identify areas for improvement and gaps in service delivery   1. Data used for decision making on areas for improvement in service delivery 2. Other (specify) 3. Don’t know |
| D26g | What challenges do you face in reviewing, interpreting, or using data?  *(Multiple responses possible)* |  | 1. Do not use data 2. Data is difficult to understand 3. Do not feel data is accurate/problems in data quality 4. Do not feel review/use of data is important 5. Lack of time for interpreting/discussing data 6. Data is not available for review/use 7. Other (specify) 8. Don’t know |

**SECTOR/CLUSTER/BLOCK MEETING**

Now I would like to talk to you about the sector/cluster review meetings held in your area

|  | How often are sector/cluster/block level review meetings held? |  | 1. Meetings never held 🡪 next module 2. Less than once a month 3. Once a month 4. Twice a month 5. Weekly   95.Other (specify)  99. Don’t know |
| --- | --- | --- | --- |
| D27a | Do you usually attend sector/cluster/block review meetings when they take place? |  | 1. Always 2. Sometimes 3. Rarely 4. Never 🡪 next module |
| D27b | Generally, who is present at the sector/cluster review/block meetings?  *(Multiple responses possible)* |  | 1. AWWs 2. ANMs 3. ASHAs 4. Lady supervisors 5. DPO 6. CDPO 7. BPM 8. ASHA Sangini 9. BCPM   10.BMO/MOIC  11. LHV  ~~12. DCPM~~  13. Any Health official  14. Basic health worker (BHW)  15. Village head/Pradhan  95.Others(Specify) |
|  | What activities take place during sector/cluster/block review meetings?  *(Multiple responses possible)* |  | 1. Update due list of beneficiaries 2. Submit monthly progress report (MPR) 3. Discuss any issues from the field 4. Receive refresher training on nutrition 5. Provide refresher training on nutrition 6. Review registers and records (e.g. pregnant listing THR registers, immunization records, etc) 7. Organize/plan upcoming activities 8. Review of growth monitoring data 9. Review data on registration of pregnant women 10. Review data on coverage of maternal health & nutrition services (e.g. early registration in 1^st^ trimester, 4 ANC, IFA & calcium supplement provision, weight check and weight gain monitoring) 11. Review data on MN counselling provided (diet counselling, counselling on IFA and calcium supplements) 12. Review data on supply chain for IFA 13. Review data on supply chain for calcium 14. Review data from MN subcenter & block cards (HMIS data based) 15. Review data from supervision checklist   95. Other (specify)  99. Don’t know |
|  | What discussions take place during sector/cluster review meetings?  *(Multiple responses possible)* |  | 1. Nothing happens 2. Review of data 3. Supervisor provides feedback from supervisory visits conducted 4. Supervisors provides refresher/training on maternal nutrition 5. Supervisor highlights progress/successes with community activites and services related to maternal nutrition 6. Supervisor highlights gaps/challenges with community activites and services related to maternal nutrition 7. FLW provides information on progress of maternal nutrition activities 8. FLW discusses challenges faced in provided maternal nutrition services 9. Discussion on IFA/calcium stock situation in the catchment area 10. Decisions on actions to improve service delivery   95. Other (specify) |
| D28a | What types of decisions/programmatic actions are made based on discussions during sector/cluster/block review meetings?  *(Multiple responses possible)* |  | 1. Nothing happens 2. Decisions on microplans for VHNDs, CBEs, home viists 3. Decisions on health and nutrition service delivery 4. Decisions/follow up on action items from previous meetings 5. Actions/prioritization related to registration and coverage based on HMIS data 6. Actions/prioritization related to quality of services especially counselling based on supportive supervision data 7. Actions/prioritization related to supply chain of IFA and calcium supplements 8. Decisions on supportive supervision visit plans 9. Other (specify) |
|  | Have you ever seen/heard about MN subcenter or block report card data?  *Prompt: Show picture of MN subcenter and/or block card* |  | 1. Yes 2. No 🡪 D32 |
|  | Are MN subcenter report cards discussed during sector/cluster review meetings? |  | 1. Yes 2. No 🡪 D32 |
|  | How is MN subcenter card data used for discussion and/or decision-making during sector/cluster review meeetings?  *(Multiple responses possible)* |  | 1. Discuss status of MN indicators in the subcenter area for each month 2. Identify areas/indicators where subcentre has progressed over time on MN 3. Identify areas/indicators where level of coverage/service delively is low 4. Prioritize areas for improvement 5. Decide on next steps to achieve improvements in identified areas 6. Other (specify) |
| D31a | Are MN block report cards discussed during block review meetings? |  | 1. Yes 2. No 🡪 D34 |
| D31b | How is MN block card data used for discussion and/or decision-making during sector/cluster review meeetings?  *(Multiple responses possible)* |  | 1. Discuss status of MN indicators in the block for each month 2. Identify areas/indicators where block has progressed over time on MN 3. Identify areas/indicators where level of coverage/service delively is low 4. Prioritize areas for improvement 5. Decide on next steps to achieve improvements in identified areas 6. Other (specify) |
|  | Have you ever seen/heard about supportive supervision checklist/quality of counseling data?  *Prompt: Show picture of checklist* |  | 1. Yes 2. No 🡪 D35 |
|  | Are data from supportive supervision checklist especially focusing on quality of counseling discussed during sector/cluster review meetings? |  | 1. Yes 2. No 🡪 D35 |
|  | How is supportive supervision/quality of counseling data used for discussion and/or decision-making during sector/cluster review meetings?  *(Multiple responses possible)* |  | 1. Quality of counselling (completeness and accuracy) on MN topics discussed 2. Identify areas/topics with high quality of counselling by FLWs 3. Identify topics where quality of counselling is low 4. Prioritize topics for improving quality of counselling on MN 5. Decide next steps to improve quality of MN counselling in block   95. Other (specify) |
|  | According to you, what are some of the challenges in using data for discussion and/or decision-making during sector/cluster review meetings?  *(Multiple responses possible)* |  | 1. Meetings do not take place regularly 2. Data is not available regularly for discussion 3. Data is not easy to use and understand 4. Required personel not present to facilitate data discussion 5. Lack of time to discuss data during meetings/too many administrative tasks 6. Do not feel it is useful to discuss data during meetings 7. Infrastructural constraints in dislaying/discussing data during meetings – overcrowded rooms, lack of adequate space/facilities, etc   95. Other (specify) |
